# The effects of intermittent fasting diet on quality of life, clinical symptoms, inflammation, and oxidative stress in overweight and obese postmenopausal women with rheumatoid arthritis: study protocol of a randomized controlled trial

**DOI:** 10.1186/s13063-024-07977-2

**Published:** 2024-03-05

**Authors:** Mahsa Ranjbar, Sakineh Shab-Bidar, Abdolrahman Rostamian, Hamed Mohammadi, Kurosh Djafarian

**Affiliations:** 1 Department of Clinical Nutrition, School of Nutritional Sciences and Dietetics, Tehran University of Medical Sciences, Tehran, Iran; 2 Department of Community Nutrition, School of Nutritional Sciences and Dietetics, Tehran University of Medical Sciences, Tehran, Iran; 3 Neuroscience Institute, Sports Medicine Research Center, Tehran University of Medical Sciences, Tehran, Iran; 4 Rheumatology Research Center, Imam Khomeini Hospital Complex, Tehran University of Medical Sciences, Tehran, Iran

**Keywords:** Rheumatoid arthritis, Fasting, Intermittent fasting

## Abstract

**Background:**

Rheumatoid arthritis (RA) is known as a chronic systemic autoimmune disorder that primarily targets synovial joints, and may cause pain and functional limitations. Studies show diet can have beneficial effects on symptoms and oxidative stress of this disease. Intermittent fasting (IF) is a dietary approach with cycles of fasting and intake. The current study aims to investigate the effect of IF on quality of life, clinical symptoms, inflammation, and oxidative stress in overweight and obese postmenopausal women with RA.

**Methods:**

The current study is a randomized clinical trial, in which 44 patients with mild to moderate severity of RA will be randomly allocated to receive either IF (*n* = 22) or the usual diet (*n* = 22) for 8 weeks. Anthropometric measures and biochemical indicators including serum concentrations of erythrocyte sedimentation rate (ESR), c-reactive protein (CRP), and total oxidant and antioxidant capacity (TOC and TAC) will be assessed at the baseline and end of the study. Also, disease severity will be assessed by Disease Activity Score-28 (DAS-28) and clinical disease activity index (CDAI), and disability index will be assessed by Health Assessment Questionnaire-Disability Index (HAQ-DI) questionnaire.

**Discussion:**

Studies show fasting has beneficial effects on inflammatory markers and results in an improvement in the health of different populations. Literature review shows it seems there is no study in this field to evaluate the effects of IF on RA patients, and they are limited to other types of fasting. However, studies show IF can have many positive effects on chronic and autoimmune diseases. Therefore, IF may have positive effects on these patients.

**Trial registration:**

IRCT20230217057441N1. Registered on 14 February 2023. https://en.irct.ir/user/trial/68669.

**Supplementary Information:**

The online version contains supplementary material available at 10.1186/s13063-024-07977-2.

## Background

Rheumatoid arthritis (RA) is a chronic and systemic autoimmune disorder that primarily affects the synovial joints. This condition can cause pain, swelling, and stiffness, leading to a decrease in mobility and quality of life, and may cause pain and functional limitations [1]. RA is a widespread inflammatory arthritis that is one of the leading causes of disability and mortality around the world [2]. It can cause many symptoms like pain, dysfunction, fatigue, and depression, which may reduce the patient’s quality of life [3], and result in a promotion in the risk of cardiovascular diseases and a decrease in life expectancy [4]. The prevalence of this disease in the world is estimated at 0.5 to 1%, which has a large variation among different populations [5]. The prevalence of RA in Iran in 2016 was reported as 0.37% [6].

The pathophysiological mechanisms of RA have not been fully understood [7]. Studies suggest that RA is related to oxidative stress, a state where the supply of reactive oxygen species increases with time and results in increased production, decreased antioxidant defense, or a combination of both mechanisms and impaired redox signaling and molecular damage control [8, 9].

Studies have stated that in middle age, lifestyle, including nutrition, and physical activity play a role in the prevention and improvement of several health problems [10–13]. Evidence shows the effects of diet and nutrients on the progress of RA [14], and the role of them as a potential tool for preventing and managing disease [15]. It is also suggested eating habits can increase the risk of disease or be a protective factor against that [16]. In recent studies, dietary interventions show significant improvements in RA patients [17, 18]. For instance, a systematic review and meta-analysis demonstrate significant improvements in the anti-inflammatory diet in the pain score of RA patients compared to the control group [19]. Also, another review showed significant beneficial long-term impacts of fasting followed by a vegetarian diet on RA patients [20].

Intermittent fasting (IF) diets have recently become popular for improvement in many chronic diseases [21]. IF is one of the types of fasting diet suggested by experts and various studies because the potential benefits of weight control, cardiovascular health, and oxidative stress reduction make it an attractive option [22]. This type of fasting refers to not consuming food or restricting its consumption in a certain period, for example, 16 h a day [23]. It seems IF can have positive effects on improving the autoimmunity in several autoimmune diseases [24, 25]. Likewise, with hormone secretion and oxidatively related gene expression, IF can protect against oxidative stress and has immunomodulatory effects [26].

Due to the pathophysiology of RA, and the autoimmune feature of this disorder, it seems IF may have beneficial effects on RA patients. Literature review showed most of the studies conducted in the field of fasting and RA have investigated total or subtotal fasting [27–30], or calorie restriction [31, 32]. To the best of our knowledge, there is no published study about the effects of IF in these patients around the world. Therefore, according to the limitations of complete fasting in the form of no food intake or restriction of calorie intake, and mentioning that this study is the first study that investigates the effects of IF in these patients, the present study will examine the effects of IF in postmenopausal, overweight, and obese women with RA in a randomized control clinical trial and parallel group manner.

## Methods

### Study design and participants

**Fig. 1:**
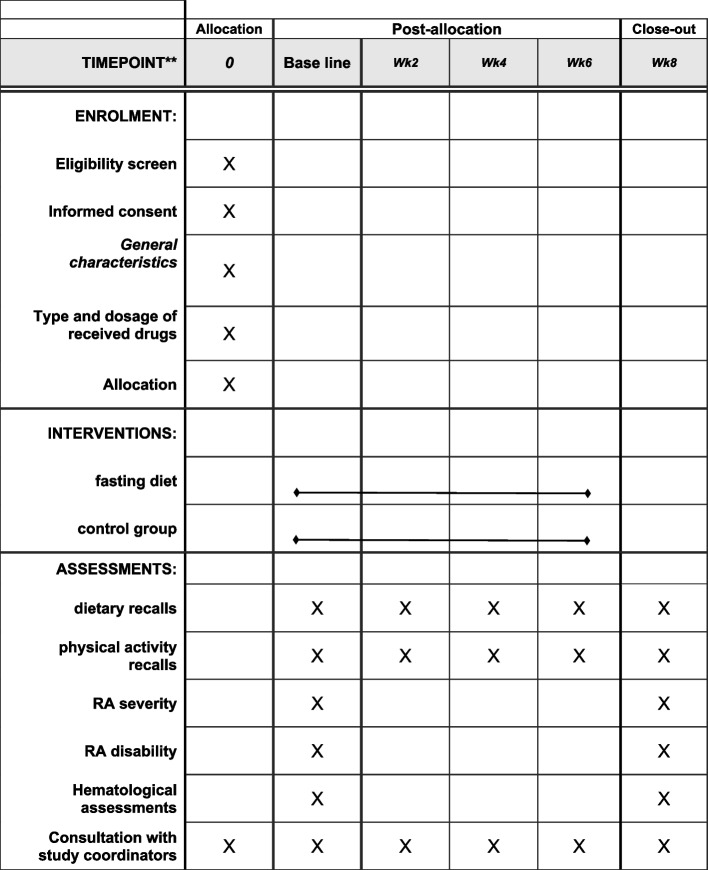
Standard Protocol Items: Recommendations for Interventional Trials (SPIRIT) chart of the enrollments and assessments during randomized controlled trials

In this randomized, controlled, parallel-group superiority trial, we compare the effects of the IF diet to the usual diet on clinical symptoms, inflammation, and oxidative stress in overweight and obese postmenopausal women who suffer from RA with the diagnosis of a rheumatologist and by the criteria of the American College of Rheumatology (ACR) over 8 weeks, with a 1:1 allocation ratio, in 2023–2024. This study will be conducted on eligible patients who will be referred to the rheumatology clinics of the Tehran University of Medical Sciences. Participants will be randomly allocated into a control group with the usual diet, and an intervention group with the IF diet. The protocol of this study was approved by the Ethics Committee of Tehran University of Medical Sciences (IR.TUMS.IKHC.REC.1401.376) and was registered at the Iranian Registry of Clinical Trials (IRCT20230217057441N1). After explaining the objectives of the study and the method of its implementation, documented informed assent is obtained from the attendees (Fig. 1).

**Fig. 2:**
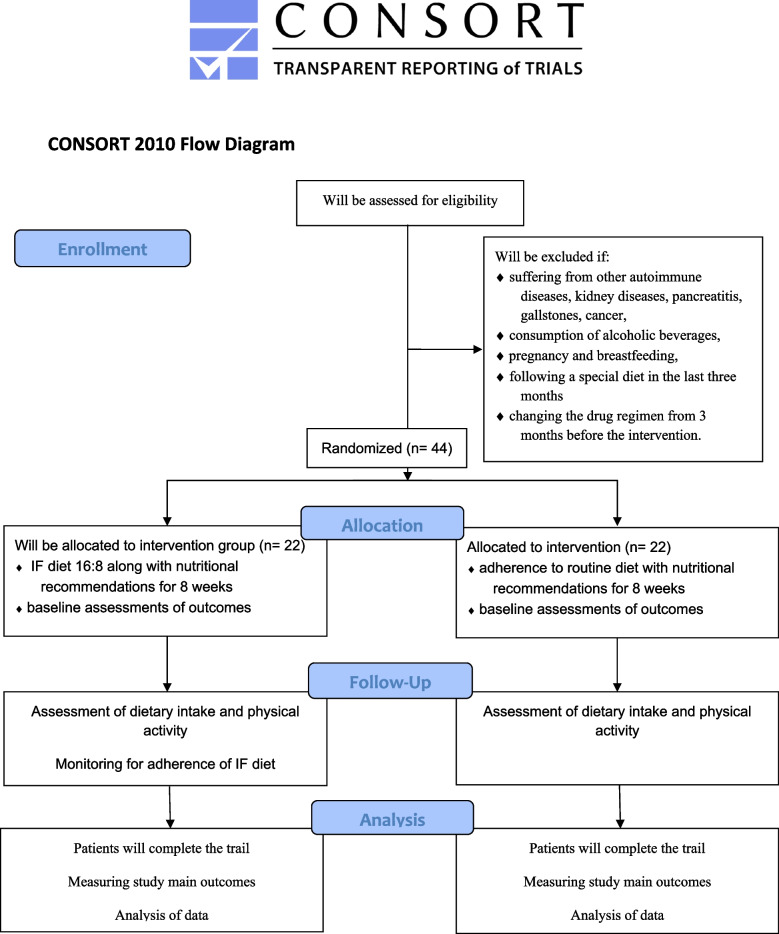
Diagram of the study design

Inclusion criteria are menopause women with a body mass index between 25 and 35 kg/m^2^, age range between 50 and 70 years, diagnosed with RA by a specialist for more than 6 months, have moderate to low RA disease activity (i.e., disease activity score <5.1), following a stable drug regimen for 3 months before the intervention, not receiving non-steroidal anti-inflammatory drugs (NSAIDs), and more than 7.5 mg of corticosteroids, and willingness to cooperate. Exclusion criteria are consumption of alcoholic beverages, suffering from other autoimmune diseases, kidney diseases, pancreatitis, gallstones, cancer, pregnancy, and breastfeeding, following a special diet in the last 3 months, changing the drug regimen from 3 months before the intervention. Figure 2 demonstrates the flow diagram of the project.

### Randomization

Participants will be randomized using stratified permuted blocks. A block size of 4 will be used, and stratification factors are the participant’s BMI (between 25 and 30 and 30 and 35 kg/m^2^) and whether they are receiving biological or non-biological drugs. People will be allocated to one of the two groups of intervention and placebo. Random allocation will be based on a random number list; the letters A and B will be assigned to an equal number of random number list. In this study, patients, doctors, and researchers are not blinded.

## Study hypothesis

We hypothesized that the IF diet has a beneficial effect on quality of life, clinical symptoms, inflammation, and oxidative stress in postmenopausal, overweight, and obese women with RA.

### Outcome measures

#### Primary outcomes

In the current study, the primary outcome is the changes in health assessment quality (HAQ) score from baseline values.

#### Secondary outcomes

Secondary outcomes are the changes in erythrocyte sedimentation rate (ESR), C-reactive protein (CRP), disease activity score in 28 joints (DAS-28), Visual Analogue Scale pain intensity (VAS-pain), level of total oxidant and antioxidant capacity (TOC and TAC), oxidative stress index (OSI), tender joint count (TJC), swollen joint count (SJC), tender-swollen joint count difference (TSJD), morning stiffness count (MS), patient global assessment of disease activity (PGA), global assessment of disease activity (EGA), and clinical disease activity index (CDAI) from baseline values.

We will evaluate both primary and secondary outcomes two times, first after the run-in period, at the beginning of the intervention, and second, 8 weeks later.

### Run-in period

Before starting the intervention, the selected people first will be entered into a 2-week run-in period to collect enough information about their food habits and intake. Then, by using a general information questionnaire about age, gender, education level, history of diseases and hospitalization, surgery, and history of taking drugs, supplements will be taken from all people. During this period, patients will complete a 24-h food record for 3 days including two working days and one day off per week, and questions will be asked about their eating habits. Before the intervention, anthropometric measures such as height, weight, waist circumference, and body composition, as well as biochemical evaluations including TOC, TAC, CRP, and ESR, will be measured and questionnaires related to the pain and inflammation will be collected. At the end of the run-in period and before the commencement of the intervention, people will be divided into blocks based on their BMI and medications received.

### Intervention

The participants in the intervention group follow the IF diet (16:8) in which they are only allowed to receive water and non-energy drinks, tea, coffee, and sugar-free gums for 16 h and 8 h in free consumption mode; they will receive food along with healthy diet recommendations for eight weeks; and the control group will receive their usual diet, along with healthy diet recommendations. To evaluate the level of adherence to the patient’s diet during the intervention, four 24-h food records (including two working days and one day off) will be collected from the patients once every 4 weeks and the physical activity will be checked every 4 weeks using the physical activity registration form and the patients will be asked to record these four physical activities in a way that includes at least two working days and two weekend days. All subjects will be asked in their physical activity not to have any change and medication and its dosage during the study compared to the beginning of the study. If there is a change in the type and dosage of the medicine received, participants will be excluded from the study. For better compliance of participants with the intervention, phone calls will be made with participants every week during the study and possible problems related to compliance with the IF diet will be investigated and necessary guidance will be given. At the end of the intervention, all the evaluations performed at the beginning of the study, including anthropometric indicators, body composition, biochemical evaluations, and questionnaires related to the amount of pain and inflammation in patients with RA, will be evaluated. If adherence to the prescribed diet is < 70% (failure to attend more than 3 phone sessions in a row, or 3 times of non-observance of the fasting diet per week for more than 2 consecutive weeks, changing the type and dosage of drugs received during the study), using new drugs and supplements and changing the dosage of the drugs being used and worsening the disease by the patients, they will be excluded from the study. An announcement to participate in this study will be printed and distributed.

### Measuring the adherence to the diet

To encourage adaptation to the IF diet and to have a healthy food intake, during the intervention, all subjects will have weekly and monthly meetings with a nutritionist so that while the intervention group follows the IF diet, all the studied subjects will use the strategies for healthy food intake. In addition, all patients will be given written and verbal instructions for healthy diet recommendations, and as mentioned, there will be no disparity between the participants in the intervention and control groups. Monitoring of the fasting diet is done through the daily interaction of the researcher and the participant in the project (for example, through telephone conversations, taking a 3-day food record). The researcher will hold weekly telephone meetings with all participants to answer questions and check compatibility with the fasting diet in the intervention group. To measure the compliance of the people in the intervention group, if they do not attend more than two phone sessions in a row, or if they do not follow the fasting diet three times a week for more than two consecutive weeks, they are considered non-compliant. In addition, the researcher provides instructions on keeping accurate records of portion sizes and food items to the participants [33].

### RA diagnosis

Diagnosing RA in patients to participate in the study will be done by a rheumatologist. Because the initial symptoms of the disease are not particularly distinctive, diagnosis of this condition can be delayed [34]. The American College of Rheumatology (ACR) has put forth criteria for the categorization of RA, which has a sensitivity of 91–94% and a specificity of 89% [35]. In 2010, the American College of Rheumatology unveiled a revolutionary new diagnostic criterion for the disease, based on which a definitive diagnosis of RA was dependent on receiving a score of 6 or more than 10. The diagnosis of the disease was largely determined by the number and type of joints affected, the levels of acute phase reactants, and the duration of the illness [36].

### Menopause

Menopause is defined as the permanent end of menstrual cycles, which is determined after a woman has gone 12 months without a period and there is no other underlying medical cause [37]. We will ask participants about the time of the beginning of menopause.

### Medications

RA patients use two drug lines to control their symptoms. The first line of these drugs, which are used to improve pain and inflammation in the acute phase of the disease in these patients, include aspirin, non-steroidal anti-inflammatory drugs, corticosteroids, and opioids [38]. The second line of drugs called disease-modifying antirheumatic drugs (DMARDs) are aimed at accelerating recovery by slowing down or stopping the progress of joint destruction and deformation, which include biological and non-biological drug groups [39]. In the current study, according to the inclusion criteria, patients should not change their drug regimen in the last 3 months and use non-steroidal anti-inflammatory drugs. Also, the use of biological and non-biological drugs is blocked in randomization.

### Adverse events

We will request that patients inform us of any adverse events that may have taken place during the study period; in case a participant experiences any rare adverse effects from the intervention or decides to discontinue the study for personal reasons, they will be removed from the study.

### Assessment of food intake

Assessment of food intake through 24-h food recording (including two working days and one day off) will be taken from participants during the intervention at the beginning, weeks 2, 4, 6, and 8. These registers will be taken by an experienced and trained expert and after the process of the data obtained from the food registers, the average intake of food items in these registers will be calculated and will be expressed as grams per day. Then, participants’ nutrient intakes will be calculated by entering these data into Nutritionist4 (N4) software.

### Assessment of physical activity

To carry out this project, physical activity records that can be used for ages 15–70 are used. Participants will fully record all their activities during the day. The start time, end time, and intensity of each type of activity will be recorded. The total duration of all participant’s recorded activities should be about 24 h. Then, the obtained data will be reported in terms of average MET-Hour per week.

### Anthropometric assessment

Anthropometric measurements including body weight, height, waist circumference, and BMI calculation (by dividing weight (kilograms) by the square of height (meters)) will be done. Body weight will be measured using a digital scale with minimal clothes and no shoes with an accuracy of 100 g. The standing height of people will be measured using a standard height meter, without shoes, and with an accuracy of 0.5 cm. A tape measure will be used to accurately measure the waist circumference to the nearest 0.5 cm, located midway between the supra iliac bone and the last rib.

### Health and disability assessment

HAQ-DI is widely regarded as the go-to tool for evaluating the functional capabilities of individuals with RA [40], and clinical variables are strongly associated with joint replacement, work disability, and mortality [41]. The reliability and validity of this questionnaire have been proven in the Iranian population [42]. HAQ was one of the first tools based on general and patient-oriented dimensions [43] and HAQ-DI is the main component of the HAQ, which was developed and validated in the late 1970s under the auspices of the Stanford Arthritis Centre [44]. HAQ-DI was developed by analyzing different questions and components from existing instruments at the time and was designed to provide a model of patient-centered outcome assessment and has an important role in many different fields including predicting successful aging, inversion pyramid therapy in RA, and investigating the risk of mortality in RA [44]. This survey consists of 20 questions divided into 8 functional categories, including dressing, grooming, rising, eating, walking, personal hygiene, lifting, and grabbing (objects) and outdoor activities. Each question is rated on a scale of 0–3, where 0 represents “no problem” and 3 represents “can’t do.”

### Assessment of inflammation and pain

VAS-pain questionnaire will be used to evaluate the pain level in patients. This tool is widely employed to assess pain levels, in which the patient is asked to rate the intensity of pain experienced measured on a 100-mm horizontal line with anchor-like descriptors in the pain field including “no pain” and mark the “worst imaginable pain” on two lines, and then measure it from the left edge (= VAS score) [45]. CDAI will be used to evaluate inflammation in these patients. To measure this index, the total scores of four criteria TJC28, SJC28, and PGA with a scale of 0 to 10 cm including “very good” and “very bad” along two horizontal lines and overall disease activity assessment (EGA) with a scale of 0 to 10 cm including “very good” and “very bad” along two horizontal lines will be calculated. Finally, the points will be classified as below [46]:

≤ 2.8 disease recovery

> 2.8 to 10 low disease activity

> 10 to 22 moderate disease activity

> 22 high disease activity

DAS-28 criterion will be used to evaluate the level of disease activity in patients. DAS28 score will be counted using TJC28, SJC28, CRP, and VAS disease activity (0–100 mm) of the patient. The following mathematical formula is used to calculate the overall score:

$$\textrm{DAS}28\textrm{- CRP}={0.56}^{\ast }\ \left(\textrm{TJC}28\right)+{0.28}^{\ast}\left(\textrm{SJC}28\right)+{0.36}^{\ast}\ln \left(\textrm{CRP}+1\right)+{0.014}^{\ast}\textrm{VAS}+0.96$$

DAS28 score can vary from 0 to 9.4 and its interpretation is according to the following data [47]:

> 5.1: High disease activity

Between 3.2 and 5.1: moderate disease activity

Between 2.6 and 3.2: low disease activity

< 2.6: disease recovery

CDAI and DAS-28 criteria have been proposed in the latest update of the American Rheumatology Association in 2019 as valid and reliable criteria for an evaluation of RA patients [48].

To evaluate the duration of morning joint stiffness, the morning stiffness duration questionnaires and the degree of stiffness perceived by the patient will be used. Morning stiffness duration questionnaires will include options of less than 15 min, less than 30 min, less than 60 min, less than 90 min, and more than 90 min. The degree of perceived stiffness will also be determined by choosing on a scale of 0 to 10 cm, including “no stiffness” and “the highest degree of stiffness” along two horizontal lines [49].

### Blood sampling and biochemical measurements

Patients will be asked to fast for 10–12 h before having 10 cc of venous blood samples collected and then placed in the laboratory for 30 min to clot the blood, in a centrifuge with 3000 per minute for 10 min to separate the serum. The serum from the blood sample will be kept in a −80 °C freezer until the tests for measuring inflammatory factors are conducted, ensuring accurate results. Subsequently, the serum levels of CRP will be determined using the enzyme-linked immunosorbent assay (ELISA) method with the help of commercial kits. To measure ESR, the Wintergreen method will be used. In this method, the amount of 109 mmol per liter of trisodium citrate is combined with 4 ml of blood. The created mixture enters the 200 mm Westergren tube. After 60 min, the height of the transparent part of the tube is read, which is the ESR [50]. The total oxidant and antioxidant capacity will be measured using colorimetric methods with a microplate spectrophotometer (Microplate Spectrophotometer, USA Biotek). Total serum antioxidant capacity will be measured by the manual FRAP (Ferric reducing the ability of plasma) method. In this method, antioxidant compounds create colored complexes by reducing ferric ions to ferric ions. FRAP values are obtained by comparing the absorbance change at 593 nm. The stoichiometric factors of Trolox to measure the tested antioxidants including α-tocopherol, ascorbic acid, and uric acid are all 2.0, and bilirubin is considered 4.0 and will be expressed as a standard unit of Trolox (mmol Trolox equivalent/L) [51]. Evaluation of total oxidant status by a spectrophotometric method based on the oxidation of ferrous ion to ferric ion in the presence of oxidant substances such as hydrogen peroxide in an acidic environment and measurement of a ferric ion with the color of xylol orange with the maximum absorption at 560 nm and it will be expressed as μmol H2O2 Equiv./L [52]. The oxidative stress index will be calculated by dividing TOC by TAC [53].

### Study sample size calculation

Considering that the primary goal in this study is to improve clinical symptoms in the IF group compared to the normal diet (HAQ score is considered as the primary outcome in this study), the number of samples required to conduct this study based on the change in HAQ, the IF group compared to the usual diet by 0.68 (a minimum clinically important difference), and considering the test power of 80%, the type I error is equal to 0.05, and the standard deviation is 0.6 was calculated for the result of 20 people in each group. We used SPSS software for calculation.

$$n=2\times \frac{{\left({z}_{1-\frac{\alpha }{2}}+{z}_{1-\beta}\right)}^2}{\Delta ^2}\ S2$$

$${n}=2\times \left[{\left(1.96+0.84\right)}^2\times \right(\left(0/{6}^2\right)\Big]/{(0.68)}^2$$

*n* = sample size in each group

*a* = type I error = 0.05

*b* = Power = 0.80

*S* ^2^ = standard deviation of the studied result (SD)

Δ^2^ = minimal clinically important difference

Therefore, considering a 10% chance of dropping out, the final sample size will be 44 people (22 people in each group) [54].

### Data management

The researchers of this study will adhere to Tehran University of Medical Sciences regulations when storing participant information. To ensure privacy, all records will be anonymized using ID numbers and any identifying participant characteristics data will be securely safeguarded in its database. Access to the login passwords will be restricted to the trial researchers only. The principal investigator entails verifying the accuracy of coding, security, and storage of data, and performing double checks of data entry and data values to ensure accuracy.

### Statistical analysis

Analyses will be performed according to the intention-to-treat analysis (ITT) method. Therefore, all participants who entered the study (regardless of whether they completed the 8-week study period) are included in the analysis. In people who will have a drop, the last data will be used. Then re-analyses will be performed based on the protocol-based analysis method (per protocol analysis), so that the changes in the results can be seen. Patients who finished the 8-week duration of the study and were visited by the researchers will be included in the per-protocol analysis. Descriptive and analytical statistics will be used for data analysis and all analyses will be done in SPSS version 24 software. The normal distribution of the variables will be checked through the scatter plot, histogram, and Shapiro-Wilk test. Variables with non-normal distribution will be reported as median (interquartile range). The mean (standard deviation) will be employed to quantify quantitative variables, while a frequency report (percentage) will be used to characterize qualitative variables. A comparison of absolute or relative frequency is done using the chi-square statistic. Between-group differences in trial results will be evaluated using a general linear mixed model for continuous variables and a chi-square test for categorical variables. Based on this, the significance will be defined in values of *P* less than 0.05 and *q* less than 0.1. Cohen’s changes will be calculated based on the mean and standard deviation, and based on that, the effect size (0.20, 0.50, and 0.80, respectively, low, medium, and high therapeutic effect) of dietary intervention will be determined. Also, a *P*-value < 0.05 will be considered statistically significant in all analyses.

### Interim analyses

Given the lack of any adverse effects observed in prior studies, no interim analysis or stopping rules are expected to be necessary for our current study of the IF diet.

*Plans to give access to the full protocol, participant-level data and statistical code*

It is possible to access the full details of the study protocol through IRCT.ir (IRCT20230217057441N1). The corresponding author can provide access to the data and participant datasets that were analyzed during the study.

### Oversight and monitoring

MR and KDj, the lead study coordinators of the study, will be the ones who conceived the study design. The Trial Steering Committee are MR, HM, and KDj. In addition, administrative and research advisors (SSb) and study physicians (AR) will also help to do the study. The dedicated committee will convene monthly to assess the progress of the study and address any financial or technical issues that may arise. Given the low-risk nature of the study, there is no need for a separate data monitoring committee. The Clinical Nutrition Department and Biochemistry Lab at Tehran University of Medical Sciences will serve as the coordinating centers for this project. All laboratory tests, such as blood sampling and serum collection, will be conducted in the Biochemistry Lab. MR will be responsible for coordinating patients’ visits and collecting the consent forms. The Data Management Team will be comprised of the project investigators (MR, KDj, and SSb).

### Frequency and plans for auditing trial conduct

There is no plan to audit the relevant tests in this study. The principal investigator should ensure that the trial steering committee and the ethics committee are kept informed of any potential risks that may arise during the course of the study.

### Plans for communicating important protocol amendments to relevant parties (e.g., trial participants, ethical committees)

In the event of any substantial changes that could affect the conduct of the trial or patient safety, we will keep the Trial Steering Committee and all participants informed and document any deviations from the trial protocol using a breach report form. Additionally, edited versions of the study protocol will be made available on the Iranian Registry for Clinical Trials (IRCT.ir) for public viewing.

## Discussion

Previous studies show the effect of IF regimes on improving performance and strong resistance to a wide range of destructive mechanisms, including those that include metabolic and oxidative stress [55]. Insulin acts as the main stimulating hormone in the satiety state, glucagon acts as the main hormone in the fasting state, and the body uses the liver’s glycogen reserves to produce energy during fasting [56]. In the IF diet, a person repeatedly experiences satiety and hunger and the onset of negative energy balance in IF is when liver glycogen reserves are depleted, insulin levels decrease, glucagon levels increase, and fatty acids are metabolized to produce energy, which is usually more than 12 h after stopping food consumption and leads to autophagy, reduction of oxidative stress, improvement of metabolism, and increase of life span [57–59]. An umbrella study of meta-analyses conducted on the results of this type of diet on various factors showed improvements in CRP, TNF, and IL-6 factors in these patients [60]. A study by Uden et al. aimed at investigating the clinical performance of individuals and blood neutrophils in patients with RA after 7 days of complete fasting was conducted on 26 affected women and the results of this study showed a significant relationship between improving the inflammatory activity of joints and increasing the bactericidal capacity of neutrophils [58]. Also, a study that was conducted in 2010. The purpose of this study was to explore the alterations in intestinal microflora in individuals with RA when following a fasting or Mediterranean diet was conducted on 50 people with RA, and patients in the fasting group received about 300 calories during 7 days and the results of this study showed a significant decrease in the average score DAS-28 in both groups. Also, this study states that more studies are needed in the field of induced changes in fasting diet in patients with RA [59]. Moreover, a study was conducted to examine the effects of fasting or a ketogenic diet on serum levels of interleukin-6 and dehydroepiandrosterone sulfate in patients with RA. The results showed in addition that fasting had a positive effect on the serum levels of the investigated cases, and menopause as a confounding variable had an effect on the results of this study [29]. As noted, a large number of studies to evaluate the relationship between fasting and RA have investigated complete fasting, no food intake, or calorie restriction [27, 29, 30]. Despite the short-term positive effects of full or partial fasting with calorie restriction, this type of fasting is an impractical long-term strategy, as it should not be sustained for more than 7 days because it causes malnutrition in patients and its short-term positive effects are fleeting compared to the chronic nature of RA. Clinical and laboratory measurements tend to show improvement within a few days of beginning a fast and worsen upon resumption of diet and intake of restricted foods. Therefore, although many studies have been conducted on the effects of complete or partial fasting with caloric restriction in patients with RA and the results of them showed the overall positive effect of fasting on the symptoms of RA, considering the limitations of complete fasting in the form of no food intake or limiting calorie intake, and mentioning that until today, no study has been conducted to investigate the effects of IF in these patients in the world and Iran, this study will be conducted.

### Strengths and limitations

This study is the first clinical trial that evaluates the effects of IF on quality of life, clinical symptoms, inflammation, and oxidative stress in overweight and obese postmenopausal women with RA. This cost-effective intervention can be readily implemented in clinical settings. if the results of this study showed improvement in symptoms in these patients, after evaluation and acceptance of the next studies, it can be used to manage and improve the symptoms of RA patients. Another strength of this study is the block matching of patients in terms of several variables that may affect the findings of the study. Also, at the start and end of the study, we will assess biochemical markers, as well as subjective and objective symptoms in patients. Compliance with the intervention will be assessed throughout the study.

The limitation of this study is we just evaluate the effects of IF in menopausal women, because of limitations in block matching and there is a need for more studies to evaluate IF in other groups of patients who suffer from RA. In addition, the potential for subjective judgments to influence the evaluation of outcome variables should be taken into consideration.

### Trial status

Ongoing, protocol version: 1; 2023-03-05: This study’s recruitment period is slated to take place from July 24, 2023, to December 2023.

### Patient and public involvement

The public and patients were not consulted during the formation of the research question, implementation of the study, recruitment of participants, execution of the study, or reporting of the results. The results of the study will be published to the study participants at the end of the study using social media.

### Supplementary Information

**Additional file 1.**

## Data Availability

Not applicable.

## Abbreviations

RA: Rheumatoid arthritis
IF: Intermittent fasting
BMI: Body mass index
NSAIDs: Nonsteroidal anti-inflammatory drugs
HAQ: Health Assessment Questionnaire
ESR: Erythrocyte sedimentation rate
CRP: C-reactive protein
DAS-28: Disease Activity Score-28
VAS-pain: Visual Analogue Scale pain intensity
TOC: Total oxidant capacity
TAC: Total antioxidant capacity
OSI: Oxidative stress index
TJC: Tender joint count
SJC: Swollen joint count
TSJD: Tender-swollen joint count difference
MS: Morning stiffness count
PGA: Patient global assessment of disease activity
EGA: Global assessment of disease activity
CDAI: Clinical disease activity index
ACR: American College of Rheumatology
RF: Rheumatoid factor
ACPA: Anti-citrullinated protein antibodies
DMARDs: Disease-modifying antirheumatic drugs
HAQ-DI: Health Assessment Questionnaire-Disability Index
ELISA: Enzyme-linked immunosorbent assay
SPIRIT: Standard Protocol Items Recommendations for Interventional Trials
